# *Coix lachryma-jobi* extract ameliorates inflammation and oxidative stress in a complete Freund’s adjuvant-induced rheumatoid arthritis model 

**DOI:** 10.1080/13880209.2019.1687526

**Published:** 2019-11-21

**Authors:** Chunfang Zhang, Wenfeng Zhang, Rongyu Shi, Bangyi Tang, Shicheng Xie

**Affiliations:** a Department of Traditional Chinese Medicine, Xiang’an Hospital of Xiamen University, School of Medicine, Xiamen University, Xiamen, China;; b Department of Orthopaedic Surgery, Yankuang Group General Hospital, Zoucheng, China;; c Department of Orthopaedics, Tancheng Hospital of Traditional Chinese Medicine, Tancheng, China;; d Medical College of Xiamen University, Xiamen, China;; e Department of Joint Surgery, Affiliated Hospital of Jining Medical University, Shandong, China

**Keywords:** Joint destruction, paw oedema, phenolic compounds, antioxidant

## Abstract

**Context:** Adlay seed [Job’s tears, *Coix lachryma-jobi* L. var. ma-yuen Stapf (Poaceae)] is a Traditional Chinese Medicine, which has been investigated to treat inflammatory diseases and rheumatism.

**Objective:** This study evaluates the ameliorative effects of adlay seed extract (ASE) in a complete Freund’s adjuvant (CFA)-induced rheumatoid arthritis (RA) rats.

**Materials and methods:** The RA Sprague–Dawley rat model was induced and randomly divided into six groups with or without ASE treatment (50, 100 or 200 mg/kg). After 28 d administration, the symptoms, biochemical parameters and molecular mechanisms were investigated.

**Results:** The values of paw oedema, PGE_2_ and MMP-3 decreased from 1.46 ± 0.04 to 0.66 ± 0.07 cm^3^, from 126.2 ± 11.48 to 79.71 ± 6.8 pg/mL and from 142.7 ± 8.36 to 86.51 ± 5.95 ng/mL, respectively; the values of body weight increased from 177.25 ± 5.94 to 205 ± 6.52 g in HASE group. In addition, treatment of ASE reduced the levels of pro-inflammatory cytokines (IL-1β, TNF-α, IL-6, MCP-1), and increased the activities of antioxidant enzyme (GSH-Px, SOD, and CAT). Furthermore, ASE could suppress the mRNA expression of COX-2 and CHI3L1 and improve the mRNA expression of CAT and GPx-1 in ankle tissues of RA rats.

**Discussion and conclusions:** For the first time, our results indicated ASE exerts anti-RA effects via inhibiting pro-inflammatory factors and alleviating oxidative stress. Our finding sheds light on the research and development of anti-RA functional foods from adlay seed.

## Introduction

Rheumatoid arthritis (RA) is a systemic and autoimmune disease characterized by the progressive degradation of cartilage, stiffness of joints and chronic synovial inflammation (Kumar et al. 2016). It affects about 1% of the total world population and damages the linings of joints, leading to joint stiffness and swelling that can ultimately result in joint deformity and cartilage destruction (Torpy et al. 2011). The pathogenesis and aetiology of RA remain not fully understood. Previous evidence indicated that oxidative stress, inflammation, reactive oxygen species (ROS) and lipid peroxidation are involved in the severity and progression of RA (Shahmohamadnejad et al. 2015). Pro-inflammatory cytokines such as interleukin-6 (IL-6), interleukin-1β (IL-1β), tumour necrosis factor-α (TNF-α) and monocyte chemotactic protein-1 (MCP-1), play a vital role in joint damage during the development of RA (Umar et al. 2014; Liu et al. 2018). In addition, increased levels of ROS and oxidative stress at the site of inflammation are essential to the progression of joint damage (Phull et al. 2018). Drug therapy, including non-steroidal anti-inflammatory drugs, disease-modifying anti-rheumatic drugs, biological therapies and glucocorticoids, remains the main therapies for RA treatment. Although drug therapy could effectively mitigate the symptoms of RA, but their clinical application is limited due to the incidence of side effects, such as gastrointestinal lesions, reproductive toxicity, infections, and hepatotoxicity (Smolen and Aletaha 2015). Therefore, novel effective while low-risk RA therapeutic strategy is urgently required. In this regard, natural medicine with effective anti-inflammatory and antioxidative effects has been proposed as an alternative therapy to alleviate the progression and complication of RA (Abd El-Ghffar et al. 2018; Zhai et al. 2018).

Adlay (Job’s tears, *Coix lachryma-jobi* L. var. ma-yuen Stapf (Poaceae)), also known as Yiyimi, is a nourishing food and an annual crop, as well as a traditional Chinese medicine, which has been used for the treatment of neuralgia, inflammatory diseases, rheumatism, osteoporosis, and as a diuretic (Yang et al. 2013). Polyphenols and polysaccharides are considered to be the major active components of adlay which possess immunological, antioxidant, and anti-inflammatory effects (Huang et al. 2009; Wang et al. 2012; Yao et al. 2015). In addition, previous study has indicated that adlay bran extract inhibited the release of inflammatory cytokines and histamines in RBL-2H3 cells (Chen et al. 2012). However, no further pharmacological studies have been reported on the anti-arthritic ability of ASE on RA.

In present study, for the first time, we investigated the protective effect of ASE against CFA-induced arthritis in Sprague–Dawley rats. In addition, we also investigated the anti-inflammatory and antioxidant effects of ASE. The objective of this work was to investigate the underlying mechanisms involved in the treatment of rheumatoid arthritis diseases.

## Materials and methods

### Plant materials and reagents

The *Coix lachryma-jobi* was purchased from Xiamen Hongyi Chinese Herbal Medicine Wholesale Company (Xiamen, China) and the seeds of plant material were derived from Jinsha village of Fujian province and harvested in July 2018, and identified by Dr Chunfang Zhang. The voucher specimen of *Coix lachryma-jobi* (No. 201806) was deposited at Pharmacy College of Xiamen University. Complete Freund’s adjuvant (CFA) and celecoxib were purchased from Sigma Aldrich (St. Louis, MO). All other chemical reagents with analytical grade were purchased from Aladdin Chemical Reagent Co., Ltd (Shanghai, China).

### Preparation of ASE

Dried seed of *Coix lachryma-jobi* powder (500 g) was extracted two times with 95% ethanol (5 L each time) at room temperature for 24 h each time. The combined extract was filtered through filter paper and the filtrate was concentrated under vacuum at 45 °C to obtain a crude extract. Then, the crude extract was suspended in water and defatted with hexane. Then, the water fraction was added into the AB-8 resin for 24 h, and then AB-8 resin column was eluted with distilled water to remove the polar constituents and then eluted by 80% ethanol. The 80% ethanol eluate was collected and concentrated under vacuum at 45 °C, and then lyophilized at −20 °C for 48 h (Huang et al. 2014).

### Analysis of the ASE by HPLC

The HPLC analysis was carried out on liquid chromatography system (Waters 2695, Milford, MA) equipped with a degasser, a quaternary pumps and photodiode array detection. A Luna C18 (150 mm × 4.6 mm, 5 μm) column maintained 30 °C, and the wavelength was recorded at 280 nm. The flow was set 0.8 mL/min. The analysis of mobile phase consisted of methanol (A) and 0.1% formic acid (B) was applied to gradient elute: 0 min, 5% A; 20 min, 60% A; 24 min, 60% A.

### Animals and induction of RA

The 6–8-week-old Sprague–Dawley rats (weighing 180–200 g) were purchased from the Experimental Animal Centre of Guangdong Province and housed under laboratory temperature of 19–24 °C, relative humidity 40–60%, and 12 h light/dark cycle. The rats were allowed freely access to water and basal diets. The experimental procedures were approved by the Animal Ethics Committee of Xiamen University (Ethics no. SYXK (Min) 2013-0006) and animals studies were performed according to National Institutes of Health Guidelines of United States (National Research Council of United States, 1996). RA was induced in rats through the intradermal administration of CFA (0.1 mL) at the palmar surface of the right hind paw on day 0 of the experiment (Abd El-Ghffar et al. 2018). The incomplete Freund's adjuvant (IFA) group of rats received intraplantar injection of IFA (0.1 mL). CFA consisted of 1.0 mg heat-killed *Mycobacterium tuberculosis* (strain H37Ra) suspended in 1 mL non-metabolizable oil, IFA contained non-metabolizable oil only. Paw swelling occurring in the right hind paw of rats was measured with vernier calliper every 7 days after RA induction. Body weight of rats were measured once a weekly after RA induction.

### Experimental design

Rats were randomly divided into seven groups of eight animals each as follows: *Control group*: rats which received the oral vehicle only (0.1% DMSO) for 28 d. *IFA group*: rats which received the oral vehicle (0.1% DMSO) for 28 d. *RA group*: RA rats which received the oral vehicle (0.1% DMSO) for 28 d. *Cel group*: RA rats which administered celecoxib (5 mg/kg/day) for 28 d (Darwish et al. 2014). *HASE group*: RA rats which administered high dose of ASE (200 mg/kg/d) for 28 d (Wang et al. 2012). *MASE group*: RA rats which administered medium dose of ASE (100 mg/kg/d) for 28 d. *LASE group*: RA rats which administered low dose of ASE (50 mg/kg/d) for 28 d. The treatments were administered consecutively for 28 d starting 1 h after CFA induction.

### Blood and ankle joint tissue sample preparation

At the end of experimental, animals were anaesthetized with diethyl ether and blood was collected from the retro-orbital sinus plexus for separation of serum by centrifugation at 4000 rpm for 15 min at 3 °C, the serum samples were collected and stored at −80 °C for further analysis of biochemical estimations. After that, all rats were sacrificed by cervical dislocation. The ankle joints were removed and homogenized after adding normal saline at a ratio of 1:9 (w/v). The homogenate was centrifuged at 4000 rpm for 15 min at 3 °C and supernatant was collected and stored at −80 °C until further analysis.

### Determination of rheumatoid biomarkers

Serum levels of matrix metalloproteinase-3 (MMP3) and prostaglandin E_2_ (PGE_2_) were measured using commercial kit (Bio-Rad, Hercules, CA) according to the manufacturer’s instruction.

### Measurement of cytokines level

Serum levels of pro-inflammatory cytokines such as IL-1β, TNF-α, IL-6 and MCP-1 were measured using commercial kit (Bio-Rad, Hercules, CA) according to the manufacturer’s guide.

### Determination of oxidative stress markers

Serum superoxide dismutase (SOD), glutathione peroxidase (GSH-Px) and catalase (CAT) levels and the serum level of malonaldehyde (MDA) were measured using commercial kit (Nanjing Jiancheng Bioengineering Institute, Nanjing, China) according to the manufacturer’s instruction.

### Quantitative real-time polymerase chain reaction (qRT-PCR) for gene expression

The mRNA expression of CHI3L1, COX-2, GPX-1 and CAT in the ankle joint tissue was measured using qRT-PCR as described previously (Huang et al. 2019; Izu et al. 2019). Total RNA was extracted from frozen ankle joint with Trizol Reagent (Takara, Dalian, China) following the manufacturer’s instructions. cDNA synthesis was performed with Frist Strand cDNA Synthesis Kit (Takara, Dalian, China). SYBR Green qPCR Master Mix kit (Thermo Scientific, Wilmington, DE) was used to quantify the mRNA levels. The qPCR was performed in triplicates, the parameter of RT-PCR amplification reaction as follows: 40 cycles of 95 °C for 10 s (denaturation) and 72 °C for 30 s (annealing/extension) using the primer sequences (Table 1). The levels of mRNA expression were normalized to endogenous control gene GAPDH.

**Table 1.** Primer sequences for quantitative real-time RNA analysis.

Genes	Forward primer	Reverse primer
CHI3L1	5′-GAGCTGCTTCCCAGATGCCC-3′	5′-CATGCCATACAGGGTTACGTC-3′
COX-2	5′-CTCTTCCGAGCTGTGCTGC-3′	5′-TGTGTTTGGGGTGGGCTTC-3′
GPX-1	5′-TGAGAAGTGCGAGGTGAATG-3′	5′-CGGGGACCAAATGATGTACT-3′
CAT	5′-CCTCAGAAACCCGATGTCCTG-3′	5′-GTCAAAGTGTGCCATCTCGTCG-3′
GAPDH	5′-CAACTTTGGCATTGTGGAAGG-3′	5′-ACACATTGGGGGTAGGAACAC-3′

### The data statistical analysis

Experimental results were reported as the means ± standard deviation (SD) and the statistical analyses were carried out using GraphPad Prism Software (GraphPad software, Inc., La Jolla, CA). The differences between groups were evaluated using one-way ANOVA followed by Tukey’s multiple comparison test. *p* < 0.05 was usually regarded as statistically significant.

## Results

### Analysis of ASE by HPLC

**Figure 1.**
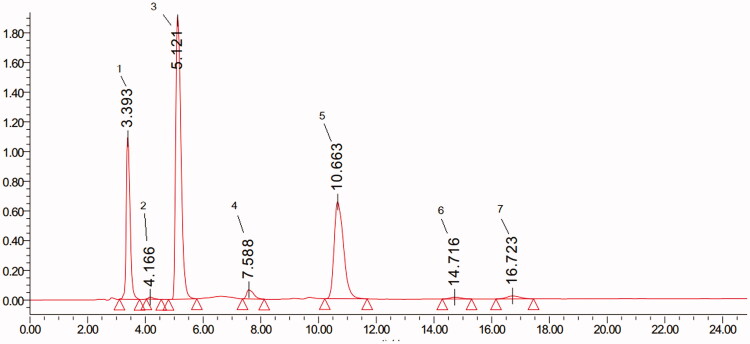
The chromatogram of adlay seed (*Coix lachryma-jobi* L.) extract (ASE) by HPLC analysis. Peak: chlorogenic acid (2), *p*-coumaric acid (3), caffeic acid (4), and ferulic acid (5).

The chromatogram of ASE indicated the presence of chlorogenic acid (2), *p*-coumaric acid (3), caffeic acid (4), and ferulic acid (5) at 280 nm as obtained with C18 (Figure 1). There are 7 peaks between 0 and 24 min, with relative area of (1) 19.50%, (2) 0.31%, (3) 47.42%, (4) 2.05%, (5) 28.84%, (6) 0.64% and (7) 1.25%.

### Effects of ASE on paw oedema and body weight in CFA-induced RA rats

**Figure 2.**
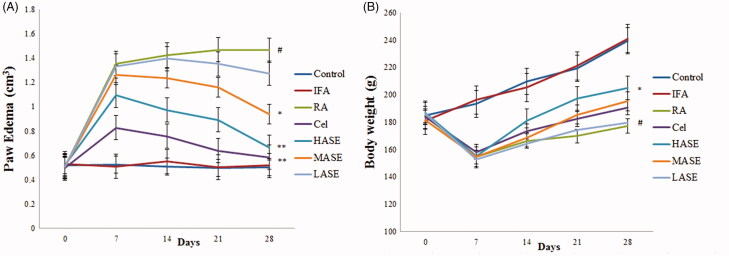
Effect of ASE on paw oedema and body weight changes in Freund’s Complete Adjuvant induced arthritis rats. (A) Paw oedema on different days of various experimental animals. (B) Average changes in body weight. Data are shown as mean ± SD (*n* = 8). Differences were analyzed using one-way analysis of variance followed by Tukey’s multiple comparison test. ^#^*p* < 0.01 versus the control group, *p < 0.05 versus the RA group, **p < 0.01 versus the RA group.

To assess the therapeutic effect of ASE on CFA-induced RA, the paw oedema and the body weight loss of rats in different groups were measured. As shown in Figure 2(A), after the intradermal administration of CFA, the model group rats developed severe paw oedema when compared with those in control group (*p* < 0.01). As expected, administration of ASE at different doses (100 and 200 mg/kg/d) obviously decreased paw oedema compared to the RA group (all *p* < 0.05). Additionally, in the RA group, there was a significant decrease of body weight compared to the control group (*p* < 0.01), which was slightly inhibited by administration of ASE (Figure 2(B)). Apparently, the therapeutic effect of ASE treatment on RA rats was comparable to that of celecoxib, especially in the HASE group (200 mg/kg/d).

### Effects of ASE on serum PGE_2_ and MMP-3 levels in CFA-induced RA rats

**Figure 3.**
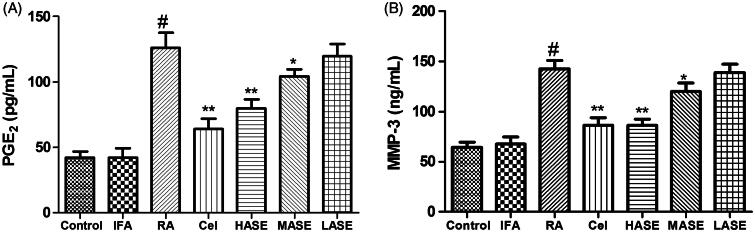
Effect of ASE on serum levels of PGE_2_ (A) and MMP-3 (B) in Freund’s Complete Adjuvant induced arthritis rats. Data are shown as mean ± SD (*n* = 8). Differences were analyzed using one-way analysis of variance followed by Tukey’s multiple comparison test. ^#^*p* < 0.01 versus the control group, *p < 0.05 versus the RA group, **p < 0.01 versus the RA group.

As shown in Figure 3, the intradermal administration of CFA obviously increased serum levels of PGE_2_ and MMP-3 as compared to the control group (*p* < 0.01). As expected, administration of ASE at different doses (100 and 200 mg/kg/d) obviously decreased serum PGE_2_ and MMP-3 levels as compared to the RA group (all *p* < 0.05). Apparently, the suppressive effect of the ASE treatment group on serum levels of PGE_2_ and MMP-3 was comparable to that of the celecoxib group, especially in the HASE group (200 mg/kg/d).

### Effect of ASE on serum pro-inflammatory cytokines in CFA-induced RA rats

**Figure 4.**
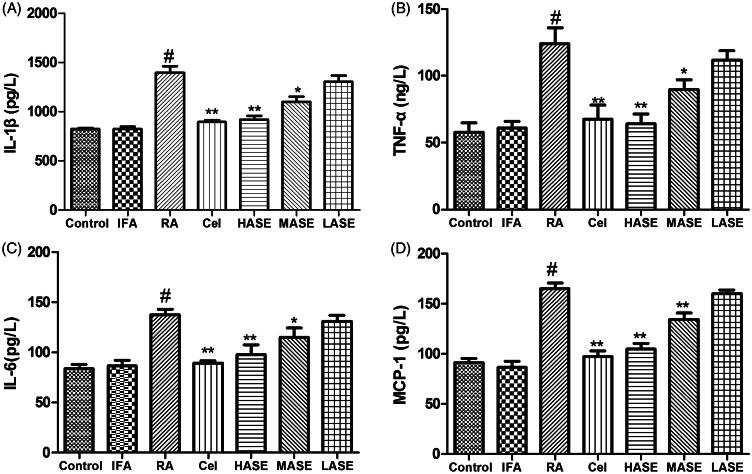
Effect of ASE on serum levels of pro-inflammatory cytokines in Freund’s Complete Adjuvant induced arthritis rats. Data are shown as mean ± SD (*n* = 8). Differences were analyzed using one-way analysis of variance followed by Tukey’s multiple comparison test. ^#^*p* < 0.01 versus the control group, *p < 0.05 versus the RA group, **p < 0.01 versus the RA group.

As shown in Figure 4, the intradermal administration of CFA obviously increased serum levels of IL-1β, TNF-α, IL-6 and MCP-1 as compared to the control group (*p* < 0.01). As expected, administration of ASE at different doses (100 and 200 mg/kg/d) obviously decreased serum IL-1β, TNF-α, IL-6 and MCP-1 levels as compared to the RA group (all *p* < 0.05). Notably, the suppressive effect of the HASE treatment group (200 mg/kg/d) on serum levels of IL-1β, TNF-α, IL-6 and MCP-1 was comparable to that of the celecoxib group.

### Effect of ASE on oxidative stress in CFA-induced RA rats

**Figure 5.**
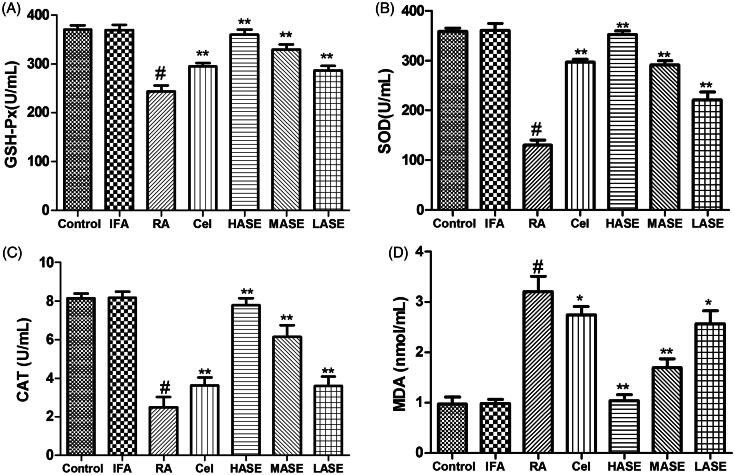
Effect of ASE on oxidative stress in Freund’s Complete Adjuvant induced arthritis rats. Data are shown as mean ± SD (*n* = 8). Differences were analyzed using one-way analysis of variance followed by Tukey’s multiple comparison test. ^#^*p* < 0.01 versus the control group, *p < 0.05 versus the RA group, **p < 0.01 versus the RA group.

As displayed in Figure 5, the intradermal administration of CFA developed an oxidative stress in the shape of decreased activities of antioxidase (GSH-Px, SOD and CAT) and increased serum MDA level of RA rats as compared to the control group (*p* < 0.01). However, these toxic effects associated with signs of arthritis were observed to be obviously improved by ASE treatment. As expected, administration of ASE at different doses (50, 100, and 200 mg/kg/d) obviously decreased serum MDA level and enhanced the activities of antioxidase as compared to the RA group (all *p* < 0.05), implying that ASE might exert anti-RA effect via assuaging oxidative stress in CFA-induced RA rats.

### Effect of ASE on mRNA expression of COX-2 (a), CHI3L1 (B), CAT (C), and GPX-1 (D) in CFA-induced RA rats

**Figure 6.**
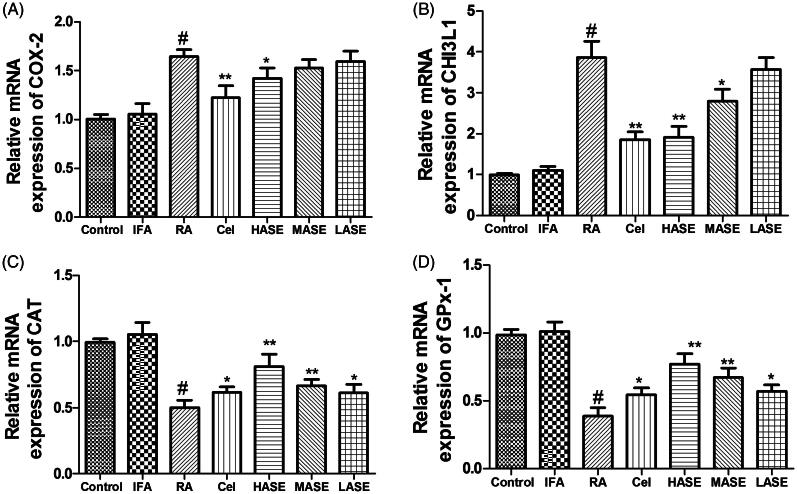
Effect of ASE on mRNA expression of COX-2 (A), CHI3L1 (B), CAT (C), and GPX-1 (D) in Freund’s Complete Adjuvant induced arthritis rats. Data are shown as mean ± SD (*n* = 8). Differences were analyzed using one-way analysis of variance followed by Tukey’s multiple comparison test. ^#^*p* < 0.01 versus the control group, *p < 0.05 versus the RA group, **p < 0.01 versus the RA group.

As shown in Figure 6, the mRNA expression levels of CAT and GPX-1 were dramatically decreased and mRNA expression levels of COX-2 and CHI3L1 were obviously increased in the RA group as compared to the control group (*p* < 0.01). HASE treatment markedly up-regulated mRNA expression levels of CAT and GPX-1 and down-regulated mRNA expression levels of COX-2 and CHI3L1 as compared to the RA group (*p* < 0.05).

## Discussion

RA is a chronic systemic inflammatory disease, which chiefly attacks the cartilage and joints. Despite the pathogenesis of RA is still unknown, several epidemiological studies have revealed pro-inflammatory cytokines and altered oxidative stress play an important role in the pathogenesis of RA (Schett and Gravallese 2012; Luczaj et al. 2016). CFA-induced RA is widely used animal model that recapitulates human RA characteristics such as joint damage, oxidative stress and increment of pro-inflammatory factors (Asquith et al. 2009; Ren et al. 2019a). In our study, CFA-induced injection resulted in an obvious joint damage manifested by oxidative stress, increment of pro-inflammatory factors and joint swelling. Interestingly, our results revealed that ASE significantly suppressed clinical sign of paw oedema, markedly reduced the oxidative stress and alleviated inflammatory response in CFA-induced RA rats. In addition, the HPLC analysis of the ASE revealed the presence of chlorogenic acid, p-coumaric acid, caffeic acid, and ferulic acid. In accordance with our findings, previous studies have indicated that treatment with *p*-coumaric acid, caffeic acid or ferulic acid attenuated joint swelling in RA rats (Zhu et al. 2018; Fikry et al. 2019; Ganesan and Rasool 2019).

Previous literatures have indicated that the increased serum levels of PGE_2_ and MMP-3 attributed to inflammatory response and oxidative stress in RA (Bae et al. 2003; Ren et al. 2019b). An increased serum MMP-3 level is a sign of cartilage destruction in the early RA (Yamanaka et al. 2000). Synovium-lining cells generate MMP-3, which plays a vital role in the degradation of type IX collagen and cartilage proteoglycans, and activates pro-collagenases (Okada et al. 1989). In the present study, the serum PGE_2_ and MMP-3 were obviously increased in CFA-induced RA rats, administration of ASE significantly reduced serum RA markers namely PGE_2_ and MMP-3.

Inflammation is a primary mechanism for CFA-induced RA rats. Infiltration of pro-inflammatory cytokines and inflammation play a vital role in the pathogenesis of RA (Schett and Gravallese 2012). The complicated network of chemokines and cytokines in the development of RA is formed through the co-adjustment in secretion and synthesis. Pro-inflammatory factors such as IL-1β, TNF-α, IL-6 and MCP-1 are considered as the primary participators which are responsible for the prolonged synovial inflammation, and eventually caused cartilage damage (Chen et al. 2019). Our results indicated that treatment of ASE obviously declined the serum levels of IL-1β, TNF-α, IL-6 and MCP-1, implying the anti-RA effect of ASE was partially via the inhibition of pro-inflammatory cytokines in CFA-induced RA rats.

CHI3L1 is a chitinase-like glycoprotein expressed in the synovial tissue, which has been positively related to inflammatory response and RA disease (Recklies et al. 2005). Previous study has reported that the serum CHI3L1 level has been considered as a biomarker for RA disease diagnose which embodies inflammatory level of RA patients (Fikry et al. 2019). COX-2 is an important enzyme participated in the generation of pro-inflammatory factors and the destruction of cartilage (Yoon et al. 2013). The present study revealed that an increase of serum pro-inflammatory cytokines accompanying with upregulation the expression of COX-2 and CHI3L1in RA rats, and similar findings were demonstrated by previous studies (Fikry et al. 2019; Ren et al. 2019a). Our results indicated that treatment of ASE obviously reduced the mRNA expression of COX-2 and CHI3L1 in RA rats, which may be attributed to the presence of phenolic compounds in ASE. In accordance with our findings, previous studies have indicated that treatment with phenolic compounds down-regulated the mRNA expression of COX-2 and CHI3L1 (Youn et al. 2017; Fikry et al. 2019).

Recent study showed that lipid peroxidation is another mechanism of the damage that happened during the RA and the levels of lipid peroxidation are increased in RA patients (Sarban et al. 2005). Organism possesses an antioxidant defence system in order to protect tissue from oxidative injure. However, the activities of antioxidant enzyme have been reported to be decreased both in RA animals and patients (Seven et al. 2008; Mateen et al. 2019). GPx-1 is generally expressed by most tissues and it protects cells against oxidative stress (Soriano-Garcia 2004). ASE has been reported to have free radical scavenging and antioxidant effects (Wang et al. 2016). In the present study, we observed that CFA resulted in an obvious depletion in GSH-Px, SOD and CAT levels and increase in MDA level. And those findings are in accordance with previous study (Ren et al. 2019a). Our findings revealed that the protective action of ASE was mediated through the improvement of antioxidant defence system and inhibiting the lipid peroxidation level.

In conclusion, the present study revealed the protective effect of ASE against paw oedema in CFA-induced RA rats. In particular, the prophylactic effects against rheumatoid arthritis were mediated via inhibiting pro-inflammatory cytokine, alleviating oxidative stress, and down-regulating the expression of COX-2 and CHI3L1. Therefore, ASE may be a potential therapeutic agent for rheumatoid arthritis in clinical application.
